# Factors associated with the 6-minute walk distance in patients with systemic sclerosis

**DOI:** 10.1186/s13075-017-1489-4

**Published:** 2017-12-15

**Authors:** Sébastien Sanges, Jonathan Giovannelli, Vincent Sobanski, Sandrine Morell-Dubois, Hélène Maillard, Marc Lambert, Céline Podevin, Nicolas Lamblin, Pascal De Groote, Jean-François Bervar, Thierry Perez, Régis Matran, Martine Rémy-Jardin, Pierre-Yves Hatron, Éric Hachulla, David Launay

**Affiliations:** 1 University of Lille, INSERM U995, LIRIC—Lille Inflammation Research International Center, F-59000 Lille, France; 2 INSERM U995, F-59000 Lille, France; 3 CHU Lille, Département de Médecine Interne et Immunologie Clinique, F-59000 Lille, France; 4 Centre National de Référence Maladies Systémiques et Auto-immunes Rares (Sclérodermie Systémique), F-59000 Lille, France; 5 Health Care Provider of the European Reference Network on Rare Connective Tissue and Musculoskeletal Diseases Network (ReCONNET), F-59000 Lille, France; 6 CHU Lille, Service de Cardiologie, F-59000 Lille, France; 7 CHU Lille, Service de Pneumologie, F-59000 Lille, France; 8 CHU Lille, Service d’Explorations Fonctionnelles Respiratoires, F-59000 Lille, France; 9 CHU Lille, Département d’Imagerie Thoracique, F-59000 Lille, France

**Keywords:** Systemic sclerosis, 6-minute walk test, Chronotropic incompetence, Exercise tolerance, Interstitial lung disease, Pulmonary hypertension

## Abstract

**Background:**

There is an ongoing debate regarding the relevance of the 6-minute walking distance (6MWD) in systemic sclerosis (SSc) assessment, widely used as a usual test in these patients as well as an outcome measure in clinical trials. In this work, we aimed to assess the associations between the 6MWD and various disease parameters in patients with SSc.

**Methods:**

Consecutive patients followed in our SSc National Reference Centre were included in this cross-sectional study if they fulfilled the 2013 American College of Rheumatology/European League Against Rheumatism criteria for SSc. Data were systematically collected during a comprehensive standardized evaluation that included a 6-minute walk test, clinical assessment, biological results, pulmonary function tests, transthoracic echocardiography, composite scores (European Scleroderma Study Group Activity Index, Medsger severity score, Health Assessment Questionnaire–Disability Index (HAQ-DI)) and treatments.

Associations of the 6MWD with various disease parameters were assessed by linear regression in univariate and multivariate analyses.

**Results:**

The study population comprised 298 patients (females 81%; mean age 58.2 ± 13.3 years; limited cutaneous SSc 82%; interstitial lung disease (ILD) 42%; pulmonary arterial hypertension (PAH) 6%). The 6MWD was significantly and independently associated with gender, age, body mass index, baseline heart rate (HR), HR variation during the test, PAH, history of arterial thrombosis and C-reactive protein levels, as well as with the HAQ-DI score in a sensitivity analysis. Muscle involvement, joint involvement and ILD were not independently associated with the 6MWD.

**Conclusions:**

During SSc, the 6MWD is independently associated with initial HR and HR variation; with PAH but not ILD, suggesting that pulmonary vasculopathy may have a greater impact than parenchymal involvement on functional limitation; and with global markers of disease activity and patient disability. These results give clinicians further insight into how to interpret the 6MWD in the context of SSc.

**Electronic supplementary material:**

The online version of this article (doi:10.1186/s13075-017-1489-4) contains supplementary material, which is available to authorized users.

## Background

Systemic sclerosis (SSc) is a severe condition classified within the connective tissue disease spectrum [1]. Over the last decades, the respiratory complications of the disease, namely pulmonary hypertension (PH) and interstitial lung disease (ILD), have become the leading cause of mortality among SSc patients [2]. One of the most important objectives when managing SSc patients is therefore to detect these complications as early as possible, to properly assess their severity and to accurately follow up therapeutic efficacy.

Among the different tools available to evaluate SSc patients, the 6-minute walk test (6MWT) is simple, noninvasive, inexpensive and reproducible, and is frequently used in daily practice [3]. Initially developed to study exercise tolerance in aviation personnel [4], its use has since been translated to the field of cardiopulmonary diseases [5], such as chronic obstructive pulmonary disease (COPD), idiopathic pulmonary fibrosis (IPF) and idiopathic pulmonary arterial hypertension (PAH). In these mono-organ conditions, the 6-minute walk distance (6MWD) has proven to be an accurate reflection of disease severity with a prognostic significance [6–8].

However, conversely to these diseases, SSc is a systemic condition associated with various extrapulmonary features (skin fibrosis, musculoskeletal pain, heart involvement, anemia) and with significant disability that can confound the interpretation of the 6MWT. In this multi-organ setting, the assumption that the 6MWD is an adequate surrogate marker for the severity of cardiopulmonary complications is no longer straightforward, and this has led clinicians to wonder what is actually being measured during this test in SSc [9]. This is of crucial importance, since the 6MWD is used in daily practice to follow up SSc patients [3], and as an outcome measure in clinical trials for SSc-PAH [10–13] and ILD [14].

Several teams have tried to answer this question but their results are conflicting [15–30]. For instance, forced vital capacity (FVC) measured during pulmonary function tests (PFT) was significantly associated with the 6MWD in some studies [19, 21, 22, 25] but not in others [16, 18, 20, 28, 29]. Similarly, associations with musculoskeletal parameters have been observed inconsistently [21, 25, 26].

To address this issue, we performed a cross-sectional study and assessed the associations between the 6MWD and various disease characteristics in a large and well-phenotyped SSc patient population.

## Methods

### Patient selection

We designed a cross-sectional study and recruited consecutive patients over 18 years old followed in the National Referral Centre for Systemic Sclerosis of Lille from November 2014 to August 2016. They were included in the study if they fulfilled the 2013 ACR/EULAR classification criteria for SSc [31]. There was no exclusion criterion.

### Data collection

Data were collected systematically by a physician in a standardized case-report form for all patients referred to our day-patient clinic. All of them underwent a comprehensive evaluation performed within the same day (except for transthoracic echocardiography (TTE) that could be performed up to 6 months before or after the 6MWT).

Patients underwent a non-encouraged 6MWT as recommended [32]. The total 6MWD (in absolute and relative values [33]), modified Borg score, peripheral oxygen saturation (SpO_2_), blood pressure (BP) and heart rate (HR) were recorded at the beginning and the end of the test. Variations of (Δ) Borg score, SpO_2_, BP and HR were defined as the difference between their final and initial values.

Other data collected included patient and SSc characteristics, clinical parameters (including modified Rodnan skin score (mRSS) and New York Heart Association (NYHA) functional class), biological results (including C-reactive protein (CRP), Nt-pro-BNP and creatinin kinase (CK) levels), TTE (including estimated systolic pulmonary artery pressure (sPAP) and left ventricle ejection fraction (LVEF)), PFT (including FVC, total lung capacity (TLC) and diffusing capacity of the lung for carbon monoxide (DLCO)), composite scores (European Scleroderma Study Group Activity Index (EScSG-AI) [34], Medsger severity score [35], Health Assessment Questionnaire—Disability Index (HAQ-DI) [36]) and treatments (for a complete list of collected data, see Additional file 1).

### Statistical analyses

Characteristics of the population were described using the mean ± standard deviation (SD) for quantitative variables, and number (percentage) for qualitative variables.

In order to study the associations between the 6MWD (in absolute value) as the dependent variable and the various explanatory variables, in a first step we conducted univariate analyses using linear regressions. Results were expressed using nonadjusted coefficients (95% confidence interval (CI)) expressed in meters. To better identify relevant explanatory variables, linear regressions were then adjusted on the main potential confounding factors (gender, age, body mass index (BMI), disease duration, smoking history and SSc subtype).

As a second step, we built a multivariate linear regression model using the following strategy. Firstly, we included a priori the six potential confounding variables already described. Secondly, we made a first selection of variables based on univariate analyses (*p* < 0.10). Thirdly, we ensured that among these variables, those of interest were present (i.e., the complications identified a priori as being able to influence the 6MWD: in particular PAH, ILD, joint and muscle symptoms, heart involvement using the left ventricle ejection fraction). Fourthly, we excluded the variables that mediated the relationship between the 6MWD and organ involvements (e.g., symptoms of dyspnea for PAH or ILD), so as not to attenuate the regression coefficients related to organ involvement variables. We also excluded collinear variables and composite scores. Fifthly, we ensured that the number of variables included in the model was consistent with the number of patients (i.e., close to one variable per 10 patients). Finally, we tested several potential interactions: between PAH or ILD and joint symptoms, muscle symptoms, smoking history or SSc subtype; and between ΔHR, initial HR and chronotropic drugs. None of the interactions tested was significant. Additionally, the HAQ-DI score was included in a separate regression model as a sensitivity analysis. Coefficients (95% CI) were expressed in meters. Regression diagnostics were performed.

All statistical analyses were performed using R software, version 3.2.5 [37]. The threshold for statistical significance was set to *p* < 0.05.

## Results

### Patient characteristics

Overall, the study population comprised 298 patients (Table 1). Most of them were middle-aged females (sex ratio male/female 0.23; mean age 58.2 ± 13.3 years) with limited cutaneous SSc (82.2%) associated with anti-centromere antibodies (56.0%). ILD (41.1%) and digital ulcers (43.0%) were the most frequent complications of the disease, while PAH (5.9%) and renal crisis (0.4%) remained rare. Patients were mainly staged in NYHA class I or II (78.7%) and displayed no major PFT alterations (mean FVC 103.5 ± 21.8%; mean DLCO 70.0 ± 19.7%). Musculoskeletal complaints included joint (42.2%) and muscle symptoms (19.2%), with mostly normal muscle enzymes (mean CK levels 93 ± 59.5 IU).

**Table 1:** Characteristics of the study population

	*N*	Value	
Demographic data			
Females, *n* (%)	298	242	(81.2)
Age at inclusion (years), mean (SD)	298	58.2	(13.3)
BMI (kg/m^2^), mean (SD)	283	24.9	(5.3)
Smoking history, *n* (%)	297	119	(40.1)
Diagnosis of SSc			
SSc subtype			
lcSSc, *n* (%)	298	245	(82.2)
dcSSc, *n* (%)	298	53	(17.8)
Disease duration at inclusion			
Since diagnosis (years), mean (SD)	298	9.1	(8.1)
Since first non-Raynaud symptom (years), mean (SD)	244	9.9	(8.5)
Since Raynaud phenomenon onset (years), mean (SD)	279	15.6	(12.2)
Immunological profile			
Anti-centromere antibodies, *n* (%)	291	163	(56.0)
Anti-topoisomerase I antibodies, *n* (%)	291	57	(19.6)
Anti-RNA polymerase III antibodies, *n* (%)	291	7	(2.4)
Anti-RNP antibodies, *n* (%)	291	15	(5.2)
Nailfold capillaroscopy			
Specific organic microangiopathy, *n* (%)	109	98	(89.9)
History of organ involvement			
Interstitial lung disease			
No ILD, *n* (%)	270	159	(58.9)
Limited ILD, *n* (%)	270	82	(30.4)
Extensive ILD, *n* (%)	270	29	(10.7)
Pulmonary arterial hypertension, *n* (%)	286	17	(5.9)
Scleroderma renal crisis, *n* (%)	285	1	(0.4)
Digital ulcers (previously or at inclusion), *n* (%)	298	128	(43.0)
Acute venous thrombosis, *n* (%)	297	33	(11.1)
Acute arterial thrombosis, *n* (%)	297	39	(13.1)
Chronic cardiovascular disease, *n* (%)	296	21	(7.1)
Clinical evaluation at inclusion			
Modified Rodnan skin score, mean (SD)	297	4.96	(5.95)
Telangiectasia, *n* (%)	275	199	(72.4)
NYHA functional class			
Class I, *n* (%)	296	132	(44.6)
Class II, *n* (%)	296	101	(34.1)
Class III, *n* (%)	296	57	(19.3)
Class IV, *n* (%)	296	6	(2.0)
Cardiovascular symptoms, *n* (%)	298	24	(8.1)
Joint symptoms, *n* (%)	296	125	(42.2)
Muscle symptoms, *n* (%)	297	57	(19.2)
6-minute walk test			
Total distance (m), mean (SD)	298	438.3	(108.1)
Total distance (% predicted), mean (SD)	290	81.6	(17.8)
Initial SpO_2_ (%), mean (SD)	280	98.1	(2.1)
Final SpO_2_ (%), mean (SD)	275	97.5	(3.3)
ΔSpO_2_ (%), mean (SD)	276	-0.58	(2.72)
Initial Borg score, mean (SD)	294	0.4	(1.1)
Final Borg score, mean (SD)	294	2.5	(2.2)
ΔBorg score, mean (SD)	293	2.1	(1.9)
Initial sBP (mmHg), mean (SD)	292	123.8	(16.6)
Final sBP (mmHg), mean (SD)	292	132.9	(18.2)
ΔsBP (mmHg), mean (SD)	291	9.2	(14.0)
Initial dBP (mmHg), mean (SD)	271	97.4	(3.4)
Final dBP (mmHg), mean (SD)	292	73.6	(10.0)
ΔdBP (mmHg), mean (SD)	291	0.95	(10.2)
Initial HR (bpm), mean (SD)	289	76.7	(12.3)
Final HR (bpm), mean (SD)	288	87.1	(15.9)
ΔHR (bpm), mean (SD)	288	10.4	(11.2)
Biological data			
Hemoglobin (g/dl), mean (SD)	291	13.4	(1.4)
ESR (mm/h), mean (SD)	258	14.8	(15.3)
CRP (mg/L), mean (SD)	293	4.0	(4.7)
Creatinin (mg/L), mean (SD)	294	8.0	(2.8)
Estimated GFR (ml/min/1.73 m^2^), mean (SD)	294	89.1	(21.5)
Elevated Nt-pro-BNP, *n* (%)	290	43	(14.8)
CK (IU), mean (SD)	289	93	(59.5)
Ferritin (ng/ml), mean (SD)	268	105.5	(136.4)
Complement activation, *n* (%)	265	9	(3.4)
Transthoracic echocardiography			
Left ventricular ejection fraction (%), mean (SD)	250	62.8	(6.7)
Left ventricular diastolic dysfunction, *n* (%)	229	124	(54.2)
Valvular heart disease, *n* (%)	244	41	(16.8)
Peak TRV (m/s), mean (SD)	98	2.62	(0.54)
RA–RV pressure gradient (mmHg), mean (SD)	206	27.4	(13.4)
Estimated sPAP (mmHg), mean (SD)	194	31.5	(13.1)
RA area (cm^2^), mean (SD)	139	15.0	(4.6)
IVC dilation and/or decreased IVC collapse, *n* (%)	210	16	(7.6)
Pericardial effusion, *n* (%)	230	5	(2.2)
Pulmonary function tests			
TLC (% predicted), mean (SD)	276	96.2	(16.4)
FVC (% predicted), mean (SD)	287	103.5	(21.8)
FEV1 (% predicted), mean (SD)	286	95.1	(21.8)
FEV1/FVC (%), mean (SD)	286	79.2	(10.7)
DLCO (% predicted), mean (SD)	285	70.0	(19.7)
KCO (% predicted), mean (SD)	278	80.9	(17.6)
Composite scores			
EScSG-AI score, mean (SD)	289	1.16	(1.2)
Medsger severity score, mean (SD)	228	4.17	(2.5)
HAQ-DI score, mean (SD)	223	0.61	(0.7)
Treatments			
Negative chronotropic drugs, *n* (%)	293	87	(29.7)
Positive chronotropic drugs, *n* (%)	293	10	(3.4)

*BMI* body mass index, *CK* creatin kinase, *CRP* C-reactive protein, *dBP* diastolic blood pressure, *dc* diffuse cutaneous, *DLCO* diffusing capacity of the lung for carbon monoxide, *EScSG-AI* European Scleroderma Study Group Activity Index, *ESR* erythrocyte sedimentation rate, *FEV1* forced expiratory volume during the first second, *FVC* forced vital capacity, *GFR* glomerular filtration rate, *HAQ-DI* Health Assessment Questionnaire—Disability Index, *HR* heart rate, *ILD* interstitial lung disease, *IU* international unit, *IVC* inferior vena cava, *KCO* diffusing coefficient for carbon monoxide, *lc* limited cutaneous, *NYHA* New York Heart Association, *RA* right atrium, *RV* right ventricle, *sBP* systolic blood pressure, *SD* standard deviation, *sPAP* systolic pulmonary arterial pressure, *SpO*_*2*_ peripheral oxygen saturation, *SSc* systemic sclerosis, *TLC* total lung capacity, *TRV* tricuspid regurgitation velocity, *Δ* variation of

During the 6MWT, patients walked a mean distance of 438.3 ± 108.1 m (81.6 ± 17.8% of the predicted value), decreased their SpO_2_ by a mean of 0.58 ± 2.72% and increased their HR by a mean of 10.4 ± 11.2 bpm. The mean Borg score was 0.4 ± 1.1 at the beginning of the test and rose to 2.5 ± 2.2 at the end.

### Associations with the 6-minute walk distance: nonadjusted and adjusted analyses

Univariate analyses revealed several associations between the 6MWD and the collected parameters (Table 2). To better identify relevant explanatory variables, these analyses were then adjusted on several potential confounding factors (gender, age, BMI, disease duration, smoking history and SSc subtype).

**Table 2:** Nonadjusted and adjusted associations between 6MWD and SSc characteristics

	Nonadjusted			Adjusted*		
	β	(95% CI)	*p*	β	(95% CI)	*p*
Male (vs female)	52.7	(21.8; 83.6)	<10^–3^	76.9	(46.4; 107.4)	<10^–3^
Age (per 1-year increment)	–3.6	(–4.5; –2.8)	<10^–6^	–3.9	(–4.8; –3.1)	<10^–6^
Smoking history (vs no history)	31.8	(6.9; 56.7)	0.01	–11.2	(–34.9; 12.6)	0.36
BMI (per 1-kg/m^2^ increment)	–4.1	(–6.4; –1.7)	<10^–3^	–3.7	(–5.7; –1.6)	<10^–3^
dcSSc (vs lcSSc)	–0.7	(–32.8; 31.5)	0.97	–40.2	(–69.7; –10.8)	0.008
Disease duration since diagnosis (per 1-year increment)	–1.8	(–3.3; –0.3)	0.02	0.7	(–0.8; 2.2)	0.38
Disease duration since first non-RP symptom (per 1-year increment)	–2.3	(–3.8; –0.7)	0.004	–0.7	(–2.3; 0.8)	0.33
Disease duration since RP onset (per 1-year increment)	–1.2	(–2.2; –0.2)	0.02	0.4	(–0.5; 1.4)	0.38
ACA (vs no ACA)	–23.4	(–48.1; 1.3)	0.06	–9.8	(–35.5; 16.0)	0.46
ATA (vs no ATA)	0.1	(–31.0; 31.3)	0.99	–2.4	(–31.5; 26.8)	0.87
ARA (vs no ARA)	47.2	(–33.2; 127.7)	0.25	14.7	(–61.6; 90.9)	0.71
Anti-RNP antibodies (vs no anti-RNP antibodies)	3.7	(–52.2; 59.5)	0.90	–22.3	(–71.0; 26.4)	0.37
Abnormal nailfold capillaroscopy (vs normal)	–18.1	(–86.7; 50.6)	0.61	–12.5	(–66.9; 41.8)	0.65
PAH (vs no PAH)	–131.8	(–183.2; –80.3)	<10^–6^	–112.0	(–155.9; –68.2)	<10^–3^
ILD (vs no ILD)			0.02			0.08
Limited ILD	16.4	(–13.0; 45.3)		14.2	(–13.8; 42.2)	
Extensive ILD	–52.3	(–95.6; –9.0)		–40.4	(–78.7; –2.1)	
DU (previously or at inclusion) (vs no DU)	13.2	(–11.6; 38.0)	0.30	–10.6	(–33.7; 12.6)	0.37
History of venous thrombosis (vs no history)	–65.0	(–103.4; –26.5)	0.001	–52.5	(–87.3; –17.6)	0.003
History of arterial thrombosis (vs no history)	–73.6	(–109.0; –38.1)	<10^–3^	–43.5	(–76.4; –10.6)	0.01
History of cardiovascular disease (vs no history)	–103.8	(–150.2; –57.5)	<10^–3^	–65.0	(–108.2; –21.8)	0.003
Modified Rodnan skin score (per 1-point increment)	–0.4	(–2.4; 1.7)	0.74	–0.9	(–3.1; 1.3)	0.42
Telangiectasia (vs no telangiectasia)	–12.4	(–41.0; 16.1)	0.39	13.4	(–12.6; 39.4)	0.31
NYHA functional class (vs class I)			<10^–6^			<10^–6^
NYHA class II	–76.1	(–100.1; –52.2)		–61.7	(–83.8; –39.6)	
NYHA class III	–132.1	(–160.8; –103.3)		–108.1	(–134.5; –81.7)	
NYHA class IV	–227.4	(–303.1; –151.7)		–216.0	(–281.6; –150.4	
Cardiovascular symptoms (vs no symptoms)	–49.8	(–94.6; –4.9)	0.03	–33.5	(–73.3; 6.2)	0.10
Joint symptoms (vs no symptoms)	–29.4	(–54.3; –4.6)	0.02	–5.8	(–28.4; 16.8)	0.61
Muscle symptoms (vs no symptoms)	–27.8	(–58.9; 3.4)	0.08	–16.1	(–44.0; 11.8)	0.26
Initial SpO_2_ (per 1% increment)	14.9	(9.2; 20.7)	<10^–6^	11.4	(6.3; 16.4)	<10^–3^
Final SpO_2_ (per 1% increment)	9.1	(5.5; 12.8)	<10^–3^	7.2	(3.9; 10.4)	<10^–3^
ΔSpO_2_ (per 1% increment)	4.7	(0.0; 9.4)	0.05	3.7	(–0.3; 7.8)	0.07
Initial Borg score (per 1-point increment)	–22.5	(–33.3; –11.7)	<10^–3^	–21.5	(–30.7; –12.3)	<10^–3^
Final Borg score (per 1-point increment)	–17.7	(–23.0; –12.4)	<10^–6^	–13.8	(–18.6; –8.9)	<10^–6^
ΔBorg score (per 1-point increment)	–15.9	(–22.1; –9.6)	<10^–3^	–10.4	(–16.3; –4.6)	<10^–3^
Initial sBP (per 1-mmHg increment)	–1.0	(–1.7; –0.2)	0.01	0.4	(–0.3; 1.2)	0.26
Final sBP (per 1-mmHg increment)	–0.6	(–1.2; 0.1)	0.11	0.8	(0.1; 1.5)	0.02
ΔsBP (per 1-mmHg increment)	0.4	(–0.5; 1.3)	0.37	0.5	(–0.2; 1.3)	0.18
Initial dBP (per 1-mmHg increment)	9.1	(5.3; 12.8)	<10^–3^	7.1	(3.9; 10.3)	<10^–3^
Final dBP (per 1-mmHg increment)	0.5	(–0.7; 1.8)	0.42	0.1	(–1.0; 1.2)	0.91
ΔdBP (per 1-mmHg increment)	0.6	(–0.7; 1.8)	0.36	0.0	(–1.0; 1.1)	0.95
Initial HR (per 1-bpm increment)	–1.9	(–2.9; –0.9)	<10^–3^	–2.0	(–2.9; –1.2)	<10^–3^
Final HR (per 1-bpm increment)	–0.1	(–0.9; 0.7)	0.86	0.1	(–0.6; 0.8)	0.85
ΔHR (per 1-bpm increment)	2.1	(1.0; 3.2)	<10^–3^	2.5	(1.6; 3.5)	<10^–6^
Hemoglobin (per 1-g/dl increment)	26.6	(18.0; 35.2)	<10^–6^	19.9	(11.5; 28.2)	<10^–3^
ESR (per 1-mm/h increment)	–2.3	(–3.1; –1.5)	<10^–6^	–2.1	(–2.8; –1.3)	<10^–6^
CRP (per 1-mg/L increment)	–5.8	(–8.4; –3.3)	<10^–3^	–5.8	(–8.0; –3.5)	<10^–3^
Creatinin (per 1-mg/L increment)	–3.6	(–8.0; 0.8)	0.11	–3.4	(–7.3; 0.5)	0.09
Estimated GFR (per 1-ml/min/1.73 m^2^ increment)	1.1	(0.6; 1.7)	<10^–3^	0.0	(–0.6; 0.5)	0.97
Elevated Nt-pro-BNP (vs normal Nt-pro-BNP)	–95.4	(–128.7; –62.1)	<10^–6^	–66.5	(–98.4; –34.6)	<10^–3^
CK (per 1-IU increment)	0.2	(0.0; 0.4)	0.11	0.2	(0.0; 0.3)	0.11
Ferritin (per 1-ng/ml increment)	0.0	(–0.1; 0.1)	0.55	0.0	(–0.1; 0.1)	0.84
Complement activation (vs no activation)	1.6	(–70.0; 73.3)	0.96	10.5	(–51.2; 72.1)	0.74
LVEF (per 1% increment)	2.1	(0.2; 4.1)	0.03	2.1	(0.4; 3.8)	0.02
LVDD (vs no LVDD)	–69.6	(–96.6; –42.7)	<10^–6^	–17.1	(–45.6; 11.4)	0.24
Valvular heart disease (vs no valvular heart disease)	–22.5	(–59.3; 14.2)	0.23	26.2	(–7.5; 59.9)	0.13
Peak TRV (per 1-m/s increment)	–62.3	(–101.0; –23.6)	0.002	–33.2	(–67.4; 1.1)	0.06
RA–RV pressure gradient (per 1-mmHg increment)	–2.5	(–3.6; –1.5)	<10^–3^	–1.8	(–2.8; –0.9)	<10^–3^
Estimated sPAP (per 1-mmHg increment)	–2.5	(–3.6; –1.4)	<10^–3^	–1.9	(–2.9; –0.9)	<10^–3^
RA area (per 1-cm^2^ increment)	–4.7	(–8.2; –1.2)	0.01	–4.6	(–8.4; –0.7)	0.02
Abnormal IVC (vs normal IVC)	–26.0	(–79.7; 27.6)	0.34	–22.5	(–68.9; 24.0)	0.34
Pericardial effusion (vs no pericardial effusion)	–57.4	(–154.7; 39.9)	0.25	–95.3	(–188.3; –2.2)	0.05
TLC (per 1% increment)	1.0	(0.3; 1.8)	0.007	1.1	(0.4; 1.8)	0.002
FVC (per 1% increment)	0.4	(–0.2; 0.9)	0.20	1.2	(0.7; 1.7)	<10^–3^
FEV1 (per 1% increment)	0.3	(–0.3; 0.9)	0.29	1.0	(0.5; 1.5)	<10^–3^
FEV1/FVC (per 1% increment)	0.8	(–0.3; 2.0)	0.16	–0.2	(–1.3; 0.9)	0.68
DLCO (per 1% increment)	1.4	(0.8; 2.0)	<10^–3^	1.5	(1.0; 2.0)	<10^–6^
KCO (per 1% increment)	0.7	(0.0; 1.5)	0.04	1.2	(0.6; 1.9)	<10^–3^
EScSG-AI score (per 1-point increment)	–16.4	(–26.2; –6.5)	0.001	–14.6	(–23.5; –5.7)	0.001
Medsger severity score (per 1-point increment)	–10.8	(–16.3; –5.4)	<10^–3^	–82.9	(–99.8; –66.0)	<10^–6^
HAQ-DI score (per 1-point increment)	–104.6	(–122.0; –87.2)	<10^–6^	–11.1	(–16.2; –6.1)	<10^–3^
Positive chronotropic drug intake (vs no intake)	–68.4	(–136.5; –0.3)	0.05	–38.7	(–98.6; 21.1)	0.21
Negative chronotropic drug intake (vs no intake)	–48.1	(–74.7; –21.4)	<10^–3^	–15.3	(–41.0; 10.5)	0.25

β coefficients expressed in meters

*Analyses adjusted on gender, age, BMI, disease duration, smoking history and SSc subtype

*6MWD* 6-minute walk distance, *ACA* anti-centromere antibodies, *ARA* anti-RNA polymerase III antibodies, *ATA* anti-topoisomerase I antibodies, *BMI* body mass index, *CI* confidence interval, *CK* creatin kinase, *CRP* C-reactive protein, *dBP* diastolic blood pressure, *dc* diffuse cutaneous, *DLCO* diffusing capacity of the lung for carbon monoxide, *DU* digital ulcers, *EScSG-AI* European Scleroderma Study Group Activity Index, *ESR* erythrocyte sedimentation rate, *FEV1* forced expiratory volume during the first second, *FVC* forced vital capacity, *GFR* glomerular filtration rate, *HAQ-DI* Health Assessment Questionnaire—Disability Index, *HR* heart rate, *ILD* interstitial lung disease, *IU* international unit, *IVC* inferior vena cava, *KCO* diffusing coefficient for carbon monoxide, *lc* limited cutaneous, *LVDD* left ventricular diastolic dysfunction, *LVEF* left ventricle ejection fraction, *NYHA* New York Heart Association, *PAH* pulmonary arterial hypertension, *RA* right atrium, *RP* Raynaud phenomenon, *RV* right ventricle, *sBP* systolic blood pressure, *sPAP* systolic pulmonary arterial pressure, *SpO*_*2*_ peripheral oxygen saturation, *SSc* systemic sclerosis, *TLC* total lung capacity, *TRV* tricuspid regurgitation velocity, *Δ* variation of

Regarding general patient and SSc characteristics, the 6MWD was associated with gender (*p* < 10^–3^), age (*p* < 10^–6^) and BMI (*p* < 10^–6^) both before and after adjustment. Associations were observed with smoking habits and disease duration in nonadjusted analysis, but did not remain significant after adjustment. Although we found no association with cutaneous subtype, immunological profile or capillaroscopic anomalies in nonadjusted analysis, adjustment on confounding variables revealed a significant association of the 6MWD with the cutaneous subtype (*p* = 0.008).

Regarding cardiopulmonary parameters, the 6MWD was significantly associated with PAH (*p* < 10^–6^), ILD (*p* = 0.02) and history of cardiovascular events (venous thrombosis *p* = 0.001; arterial thrombosis *p* < 10^–3^) in nonadjusted analysis. Association with ILD was no longer significant after adjustment (*p* = 0.08). We also found significant associations of the 6WMD with NYHA functional class (*p* < 10^–6^), elevated Nt-pro-BNP levels (*p* < 10^–6^), echocardiographic markers of PH (estimated sPAP *p* < 10^–3^; right atrium area *p* = 0.01) and left-heart dysfunction (LVEF *p* = 0.03), and PFT results (TLC *p* = 0.007; DLCO *p* < 10^–3^) in nonadjusted analysis. These associations remained significant after adjustment.

Other cardiopulmonary parameters measured during the 6MWT also displayed significant associations with the total distance, especially initial HR (*p* < 10^–3^) and ΔHR (*p* < 10^–3^), both before and after adjustment. Significant associations with chronotropic drug intake were observed only in nonadjusted analysis.

Regarding extracardiopulmonary parameters, we observed an association with articular involvement (joint symptoms *p* = 0.02) that became nonsignificant after adjustment on confounding variables. Similarly, there was no association with markers of muscular (muscle symptoms *p* = 0.08; CK levels *p* = 0.11) and skin (mRSS *p* = 0.74; digital ulcers *p* = 0.30) involvements in nonadjusted analysis.

Regarding global indicators of disease activity and severity, the 6MWD was significantly associated with hemoglobin (*p* < 10^–3^), CRP (*p* < 10^–3^), EScSG-AI (*p* = 0.001) and Medsger severity scores (*p* < 10^–3^), both before and after adjustment. We also found a markedly large association of the 6MWD with the HAQ-DI score (*p* < 10^–3^) in both analyses.

### Associations with the 6-minute walk distance: multivariate models

Based on the results of our nonadjusted and adjusted analyses, we built a multivariate model to determine the factors independently associated with the 6MWD (Table 3).

**Table 3:** Multivariate regression model assessing the associations between 6MWD and SSc characteristics

	β	(95% CI)	*p*
Male (vs female)	58.9	(22.6; 95.2)	0.002
Age (per 1-year increment)	–2.8	(–3.9; –1.7)	<10^–3^
Disease duration since diagnosis (per 1-year increment)	–0.7	(–2.5; 1.0)	0.42
BMI (per 1-kg/m^2^ increment)	–2.7	(–5.1; –0.2)	0.03
Smoking history (vs no history)	–8.2	(–33.9; 17.5)	0.53
History of venous thrombosis (vs no history)	–10.8	(–48.6; 26.9)	0.57
History of arterial thrombosis (vs no history)	–39.6	(–77.9; –1.4)	0.04
dcSSc (vs lcSSc)	–22.7	(–57.6; 12.2)	0.20
PAH (vs no PAH)	–79.2	(–129.7; –28.8)	0.002
ILD (vs no ILD)			0.15
Limited ILD	22.0	(–6.9; 50.9)	
Extensive ILD	–14.7	(–55.8; 26.4)	
Joint symptoms (vs no symptoms)	–16.5	(–41.6; 8.6)	0.20
Muscle symptoms (vs no symptoms)	–7.5	(–40.6; 25.7)	0.66
Hemoglobin (per 1-g/dl increment)	9.2	(–0.8; 19.1)	0.07
CRP (per 1-mg/L increment)	–4.3	(–6.7; –1.9)	<10^–3^
Estimated GFR (per 1-ml/min/1.73 m^2^ increment)	0.2	(–0.4; 0.9)	0.46
Initial HR (per 1-bpm increment)	–1.1	(–2.2; –0.1)	0.03
ΔHR (per 1-bpm increment)	2.8	(1.8; 3.8)	<10^–6^
LVEF (per 1% increment)	1.2	(–0.5; 2.9)	0.15
Positive chronotropic drug intake (vs no intake)	14.2	(–44.3; 72.6)	0.63
Negative chronotropic drug intake (vs no intake)	–25.3	(–52.3; 1.7)	0.07

*N* = 192. *R* ^2^ = 0.59, adjusted *R* ^2^ = 0.54

β coefficients expressed in meters

*6MWD* 6-minute walk distance, *BMI* body mass index, *CI* confidence interval, *CRP* C-reactive protein, *dc* diffuse cutaneous, *GFR* glomerular filtration rate, *HR* heart rate, *ILD* interstitial lung disease, *lc* limited cutaneous, *LVEF* left ventricle ejection fraction, *PAH* pulmonary arterial hypertension, *SSc* systemic sclerosis, *Δ* variation of

**Fig. 1:**
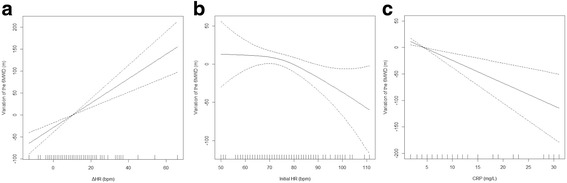
Adjusted associations in a generalized additive model. Regression splines representing the adjusted associations in a generalized additive model between the 6-minute walk distance (6MWD) and (**a**) variation of heart rate (ΔHR) during the test, (**b**) initial heart rate (HR) at the beginning of the test and (**c**) C-reactive protein (CRP) levels. *y* axis corresponds to variation of 6MWD as a function of the explanatory variables. Dashed lines correspond to 95% CI of the spline

Parameters that were significantly and independently associated with 6MWD were gender (β = 58.9 (22.6; 95.2) m for men vs women, *p* = 0.002), age (β = –2.8 (–3.9; –1.7) m per 1-year increment, *p* < 10^–3^), BMI (β = –2.7 (–5.1; –0.2) m per 1-kg/m^2^ increment, *p* = 0.03), history of arterial thrombosis (β = –39.6 (–77.9; –1.4) m vs no history, *p* = 0.04), PAH (β = –79.2 (–129.7; –28.8) m vs no PAH, *p* = 0.002), CRP (β = –4.3 (–6.7; –1.9) m per 1-mg/L increment, *p* < 10^–3^), initial HR (β = –1.1 (–2.2; –0.1) m per 1-bpm increment, *p* = 0.03) and ΔHR (β = 2.8 (1.8; 3.8) m per 1-bpm increment, *p* < 10^–6^) (Fig. 1). This model accounted for 59% of the variance of the 6MWD.

A sensitivity analysis including the HAQ-DI score revealed its independent association with the 6MWD (β = –77.1 (–101.3; –53.0) m per 1-unit increment, *p* < 10^–6^) (Additional file 2).

## Discussion

In this cross-sectional study, we tried to identify the factors associated with the 6MWD in a large and fully characterized population of SSc patients. Our results can be summarized as follows: aside from demographic parameters, we observed significant and independent associations of the 6MWD with ΔHR and resting HR, with PAH and history of arterial thrombosis, and with CRP levels; we found no independent association of the 6MWD with ILD or musculoskeletal involvement; and in a sensitivity analysis, the HAQ-DI score was also independently associated with the 6MWD.

Although literature data are conflicting, the results of our univariate analyses mostly confirm findings from previous works, which observed associations of the 6MWD with age, BMI and cardiopulmonary parameters (especially PFT and TTE), but no major influence of musculoskeletal involvement [15–30]. Multivariate analyses from previous studies have also established age [16], gender [18], HAQ scores [21] and CRP [19] as independent factors associated with the 6MWD. Independent associations with dyspnea [16], initial and final SpO_2_ [18], initial Borg score [18] and DLCO [21, 28] were also reported, but since our goal was to identify associations with specific organ involvements rather than their mediators, we chose not to include these variables in our multivariate model.

### Association of the 6MWD with ΔHR

Association between the 6MWD and ΔHR has never been studied previously in SSc. Interestingly, similar findings were made in IPF [38] and PAH [39] patients, where the variation in HR was found to be an important factor associated with the 6MWD as well as a valuable prognostic marker.

Interpretation of this result in the context of the 6MWT is challenging. On the one hand, the lower increase in HR observed in some SSc patients could simply indicate lower exercise intensity due to limited functional capacities or a lack of motivation [40]. On the other hand, it could also reflect an actual impairment of the chronotropic response to exercise. Chronotropic incompetence, defined as the inability to increase HR above 80% of its predicted peak value during a maximal exercise test, is a major cause of exercise intolerance in various cardiopulmonary diseases [40, 41]. It is a common finding in patients with heart diseases (sick sinus syndrome, atrial fibrillation, ischemic heart disease, chronic heart failure), neurological disorders (through autonomic dysfunction) or specific medication intakes (such as β-blockers and nondihydropyridine calcium-channel blockers) [40, 41].

Since the 6MWT is a submaximal exercise test, the diagnosis of chronotropic incompetence cannot be made here with certainty; and since detailed patient comorbidities are not available, the causes for the impaired chronotropic response in our population cannot be investigated fully. However, it is interesting to note that negative chronotropic drug intake was not independently associated with the 6MWD in our patients, making the hypothesis of a medication-induced chronotropic incompetence unlikely. Conversely, SSc is frequently associated with autonomic dysfunction [42] (especially in terms of HR regulation [43]) and with a specific myocardial disease [1], both of which could induce chronotropic incompetence.

Overall, this result suggests that an impaired chronotropic response could be an important factor associated with exercise intolerance in SSc. Interestingly, Someya et al. [44] showed in a previous work that the HR at the end of the 6MWT was higher in SSc patients than in controls to try and compensate for a decreased stroke volume. Thus, it seems reasonable to assume this compensation mechanism would be impaired in SSc patients with chronotropic incompetence, resulting in lower exercise capacities. In any case, this finding warrants further investigations, using maximal incremental exercise tests.

### Association of the 6MWD with resting HR

Interestingly, we found that both ΔHR and resting HR are independently associated with the 6MWD. This result suggests that, during SSc, exercise capacities are conditioned not only by the patient’s ability to increase his or her HR during exercise, but also by his or her baseline HR value.

Only two studies have so far tested the association of the 6MWD with resting HR in SSc and they yielded discrepant results [19, 29]. Our observation is supported by similar findings in patients with nonidiopathic PAH and chronic heart failure [39, 45].

Here again, interpretation of this result in the context of our study is difficult. Heart rate is tightly regulated by the autonomous system in order to adapt cardiac output to situations of stress or exercise [41]. Thus, the reduction of physical performance in SSc patients with increased resting HR could be suggestive of advanced autonomic dysfunction (loss of balance between sympathetic and vagal basal tones) and/or severe myocardial disease (compensating mechanism for a decreased resting stroke volume), as implied by our previous result. Alternatively, this could also indicate global deconditioning or performance anxiety.

Either way, further studies are needed to better appreciate the pathophysiological implications of this result.

### Association of the 6MWD with PAH but not with ILD

Our multivariate analysis also revealed a significant and independent association of the 6MWD with PAH, but not with ILD. Interestingly, all previous studies that tested the relation between the 6MWD and PH consistently found a shorter walked distance in PH patients [21, 30] or an association with echocardiographic markers of PH [16, 19, 24, 29] (with only one exception [18]). Conversely, data regarding the influence of ILD on the 6MWD are more conflicting, with some studies observing shorter 6MWD in SSc-ILD patients [16, 30] and others reporting no significant difference [20, 21]. However, accurate comparison with previous works is challenging because of heterogeneity in design and population (notably in PH diagnosis, ILD definition and severity).

It is interesting to note that our result is in line with several data pleading for a predominant importance of pulmonary hemodynamic alterations over lung parenchymal involvement. In patients with IPF and PH, the 6MWD is significantly associated with hemodynamic parameters measured during right heart catheterization (RHC), but not with lung volumes assessed by spirometry [46]. In SSc-ILD patients, gas transfer alteration has a stronger prognostic value than lung volume reduction [47, 48].

However, if this result confirms that PAH contributes to impaired exercise capacities during SSc, we also observed in a previous work that the 6MWD correlated poorly with RHC parameters in SSc-PAH patients [49]. Taken together, these results suggest an impact of confounding extracardiopulmonary factors on the walked distance.

### Association of the 6MWD with HAQ-DI score

Another interesting finding of our study is the strong association observed between the 6MWD and the HAQ-DI score.

Several teams have already reported similar results, both in univariate and multivariate regressions [21, 23]. Remarkably, in a previous work, Chow et al. [50] showed that the HAQ-DI score was not associated with parameters of cardiopulmonary severity in SSc patients with PAH. This implies that the patient perception of disability and functional limitation may not be entirely explained by the actual severity of the disease. Moreover, the 6MWD has been shown to be associated with scales of depression, anxiety and quality of life in several cardiorespiratory diseases [45, 51].

Overall, these results suggest that if the 6MWD is influenced to some extent by the objective severity of organ involvements, it is also greatly conditioned by the patient’s subjective impression of disability and quality of life impairment.

### Association of the 6MWD with CRP

An independent association between CRP levels and the 6WWD was observed in our population. A similar result was also found in previous work by Schoindre et al*.* [19] both in univariate and multivariate regressions, but not by Deuschle et al*.* [21]. Interestingly, CRP levels are associated with the EScSG-AI score, Medgser severity score and HAQ-DI score and with poorer prognosis in SSc patients [52]. In line with our prior results, this observation could suggest that the 6MWD also captures the overall disease activity and severity.

### No major influence of the musculoskeletal involvement on the 6MWD

Contrary to a common idea [9], it is interesting to note that musculoskeletal involvement is not independently associated with the 6MWD in our population. However, we did not consider which sites were involved in patients with joint or muscle symptoms. Therefore, we could not test the association between the 6MWD and lower limb involvement, which could contribute to explain this negative result. The effect of joint and muscle symptoms has been suggested previously in univariate analyses [21, 25, 26] but has never been tested in multivariate regression. Interestingly, quadricipital strength and joint involvement have been found to correlate both with the 6MWD and with the HAQ-DI score in SSc patients [25, 26, 53], which suggests that this score may also account for the musculoskeletal-induced disability impacting on the 6MWD.

Our study draws strength from a large sample size and an important number of variables collected.

Our study also has some limitations. We did not perform incremental exercise testing to correlate the 6MWD with objective physiological parameters collected during effort. Similarly, we did not study autonomic dysfunction to help explain the results observed with HR variation and ΔHR.

In our multivariate regression, we chose to include only parameters relating to organ involvement (e.g., PAH and ILD) and exclude variables mediating them (e.g., NYHA class, PFT results and TTE data). Indeed, we believed that inserting the latter in our model could mask interesting associations of the 6MWD with organ involvement.

## Conclusion

Our work showed that, aside from demographic parameters, ΔHR, resting HR, PAH, history of arterial thrombosis, CRP levels and HAQ-DI score are important factors associated with the 6MWD in SSc, while pulmonary interstitial and musculoskeletal involvements seem to have no major influence. This suggests that the 6MWD should be interpreted not only as a marker of cardiorespiratory severity, but also as a global assessment of disease activity and patient disability. Further studies are warranted to investigate the possibility of a chronotropic incompetence as a cause for exercise intolerance in SSc patients.

## Additional files

Additional file 1: presents additional methods, a complete list of data collected during the study and a definition of each collected item (DOCX 39 kb)

Additional file 2: is **Table S1.** presenting results of sensitivity analysis with inclusion of the HAQ-DI score in our multivariate regression model assessing the associations between 6MWD and SSc characteristics (DOCX 33 kb)

## Abbreviations

6MWD: 6-minute walk distance
6MWT: 6-minute walk test
ACR: American College of Rheumatology
BMI: Body mass index
BP: Blood pressure
CK: Creatinin kinase
COPD: Chronic obstructive pulmonary disease
CRP: C-reactive protein
DLCO: Diffusing capacity of the lung for carbon monoxide
EScSG-AI: European Scleroderma Study Group Activity Index
EULAR: European League Against Rheumatism
FVC: Forced vital capacity
HAQ-DI: Health Assessment Questionnaire—Disability Index
HR: Heart rate
ILD: Interstitial lung disease
IPF: Idiopathic pulmonary fibrosis
LVEF: Left ventricle ejection fraction
mRSS: Modified Rodnan skin score
NYHA: New York Heart Association
PAH: Pulmonary arterial hypertension
PFT: Pulmonary function tests
PH: Pulmonary hypertension
RHC: Right heart catheterization
sPAP: Systolic pulmonary artery pressure
SpO_2_: Peripheral oxygen saturation
SSc: Systemic sclerosis
TLC: Total lung capacity
TTE: Transthoracic echocardiography
Δ: Variation of
