# Supplementary material for: Factors associated with the 6-minute walk distance in patients with systemic sclerosis
Source: Arthritis Res Ther. 2017 Dec 15;19:279. doi: 10.1186/s13075-017-1489-4 (PMC5732461; doi:10.1186/s13075-017-1489-4)
Supplement: Supplementary file 1 — presents additional methods, a complete list of data collected during the study and a definition of each collected item (DOCX 39 kb) [file 13075_2017_1489_MOESM1_ESM.docx]

**ADDITIONAL METHODS**

**Data collection**

Data were prospectively collected for each patient during a comprehensive evaluation performed within a day (except for transthoracic echocardiography (TTE) that could be performed up to 6 months before or after the test).

Patients underwent a non-encouraged 6-minute walk test (6MWT), performed as recommended by the American Thoracic Society (ATS) [1]. Total 6-minute walk distance (6MWD) (measured in absolute value, with calculation of the relative value as previously described [2]), modified Borg score, peripheral oxygen saturation (SpO_2_), blood pressure (BP) and heart rate (HR) were recorded at the beginning and the end of the test. Variation of Borg score (ΔBorg), SpO_2_ (ΔSpO_2_), systolic BP (ΔsBP), diastolic BP (ΔdBP) and HR (ΔHR) were defined as the difference between the value at the end of the 6MWT and the value at its beginning for each parameter.

A global evaluation also recorded the following data:

- Demographics: age, sex, body mass index (BMI) and smoking history (yes/no)
- SSc characteristics: cutaneous subset according to Leroy’s classification [3], disease duration, immunological profile, presence of a specific organic microangiopathy on nailfold capillaroscopy
- Main organ involvements and relevant medical history: pulmonary arterial hypertension (PAH) (defined as hemodynamically-proven pre-capillary group 1 pulmonary hypertension [4]), interstitial lung disease (ILD) (defined by the presence of one or more of the following features on a chest high-resolution CT-scan: isolated ground-glass opacities, honeycombing with concurrent areas of ground-glass attenuation, and traction bronchiectasis and/or bronchiolectasis [5]; and staged according to Goh’s criteria [6]), digital ulcers (DU) (previously and/or at presentation), history of scleroderma renal crisis (SRC), history of acute venous thrombosis (deep vein thrombosis, pulmonary embolism) or arterial thrombosis (myocardial infarction, ischemic stroke, acute limb ischemia), history of chronic cardiovascular disease (coronary heart disease, lower extremity peripheral artery disease)
- Clinical assessment: modified Rodnan skin score (mRSS), telangiectasias, New York Heart Association (NYHA) functional score, cardiovascular symptoms (chest pain, palpitations, syncope), joint symptoms (pain or swelling), muscle symptoms (pain or weakness)
- Biological data: hemoglobin, erythrocyte sedimentation rate (ESR), C-reactive protein (CRP), creatinin and glomerular filtration rate (GFR) estimated by the Modification of Diet in Renal Disease (MDRD) equation, Nt-pro-BNP, creatinin kinase (CK), ferritin, serum complement (total hemolytic activity (CH50), complement fraction 3 (C3) and 4 (C4))
- Transthoracic echocardiography (TTE): left ventricular ejection fraction (LVEF), left ventricular diastolic function, aortic and mitral valves status, peak tricuspid regurgitation velocity (TRV), right atrium (RA)-right ventricle (RV) pressure gradient, estimated systolic pulmonary artery pressure (sPAP), RA area, inferior vena cava (IVC) diameter and collapse, pericardial status
- Pulmonary function tests (PFT): total lung capacity (TLC), forced vital capacity (FVC), forced expiratory volume during the first second (FEV1), FEV1 to FVC ratio (FEV1/FVC), diffusing capacity of the lung for carbon monoxide (DLCO), diffusing coefficient for carbon monoxide (KCO)
- Composite scores: European Scleroderma Study Group Activity Index (EScSG-AI) [7], Medsger severity score [8], Health Assessment Questionnaire-Disability Index (HAQ-DI) [9]
- Treatments: negative chronotropic drugs (β-blockers, non-dihydropyridine calcium-channel blockers, other antiarrhythmic drugs, ivabradine, cholinesterase inhibitors used for cognitive disorders), positive chronotropic drugs (β2-agonists, α1-antagonists)

**References**

1. ATS Committee on Proficiency Standards for Clinical Pulmonary Function Laboratories. ATS statement: guidelines for the six-minute walk test. Am. J. Respir. Crit. Care Med. 2002;166:111–7.

2. Enright PL, Sherill DL. Reference equations for the six-minute walk in healthy adults. Am. J. Respir. Crit. Care Med. 1998;158:1384–7.

3. LeRoy EC, Black C, Fleischmajer R, Jablonska S, Krieg T, Medsger TA, et al. Scleroderma (systemic sclerosis): classification, subsets and pathogenesis. J. Rheumatol. 1988;15:202–5.

4. Authors/Task Force Members:, Galiè N, Humbert M, Vachiery J-L, Gibbs S, Lang I, et al. 2015 ESC/ERS Guidelines for the diagnosis and treatment of pulmonary hypertension: The Joint Task Force for the Diagnosis and Treatment of Pulmonary Hypertension of the European Society of Cardiology (ESC) and the European Respiratory Society (ERS)Endorsed by: Association for European Paediatric and Congenital Cardiology (AEPC), International Society for Heart and Lung Transplantation (ISHLT). Eur. Heart J. 2015;

5. Desai SR, Veeraraghavan S, Hansell DM, Nikolakopolou A, Goh NSL, Nicholson AG, et al. CT Features of Lung Disease in Patients with Systemic Sclerosis: Comparison with Idiopathic Pulmonary Fibrosis and Nonspecific Interstitial Pneumonia. Radiology. 2004;232:560–7.

6. Goh NSL, Desai SR, Veeraraghavan S, Hansell DM, Copley SJ, Maher TM, et al. Interstitial lung disease in systemic sclerosis: a simple staging system. Am. J. Respir. Crit. Care Med. 2008;177:1248–54.

7. Valentini G, Rossa AD, Bombardieri S, Bencivelli W, Silman AJ, D’Angelo S, et al. European multicentre study to define disease activity criteria for systemic sclerosis. II. Identification of disease activity variables and development of preliminary activity indexes. Ann. Rheum. Dis. 2001;60:592–8.

8. Medsger TA, Bombardieri S, Czirjak L, Scorza R, Rossa AD, Bencivelli W. Assessment of disease severity and prognosis in systemic sclerosis. Clin. Exp. Rheumatol. 2003;21:S42–S46.

9. Fries JF, Spitz P, Kraines RG, Holman HR. Measurement of patient outcome in arthritis. Arthritis Rheum. 1980;23:137–45.
