# Supplementary material for: Factors associated with the 6-minute walk distance in patients with systemic sclerosis
Source: Arthritis Res Ther. 2017 Dec 15;19:279. doi: 10.1186/s13075-017-1489-4 (PMC5732461; doi:10.1186/s13075-017-1489-4)
Supplement: Supplementary file 2 — is Table S1. presenting results of sensitivity analysis with inclusion of the HAQ-DI score in our multivariate regression model assessing the associations between 6MWD and SSc characteristics (DOCX 33 kb) [file 13075_2017_1489_MOESM2_ESM.docx]

**ADDITIONAL TABLE**

**Additional Table 1. Sensitivity analysis with inclusion of the HAQ-DI score in our multivariate regression model assessing the associations between 6MWD and SSc characteristics.**

|  | **β** | **[95%CI]** | ***p*** |
| --- | --- | --- | --- |
| **Male** (*vs*. female) | 46,5 | [9,3; 83,7] | 0,01 |
| **Age** (per 1-year increment) | -2,6 | [-3,8; -1,4] | <10^-3^ |
| **Disease duration since diagnosis** (per 1-year increment) | 0,3 | [-1,7; 2,2] | 0,79 |
| **BMI** (per 1-kg/m^2^ increment) | -2,3 | [-4,8; 0,2] | 0,07 |
| **Smoking history** (*vs*. no history) | -5,2 | [-32,3; 22,0] | 0,71 |
| **History of venous thrombosis** (*vs*. no history) | 11,1 | [-31,2; 53,4] | 0,60 |
| **History of arterial thrombosis** (*vs*. no history) | -16,3 | [-56,6; 23,9] | 0,42 |
| **dcSSc** (*vs*. lcSSc) | -18,1 | [-53,8; 17,7] | 0,32 |
| **PAH** (*vs*. no PAH) | -44,0 | [-99,7; 11,8] | 0,12 |
| **ILD** (*vs*. no ILD) |  |  | 0.60 |
| Limited ILD | -1,6 | [-45,4; 42,3] |  |
| Extensive ILD | 14,3 | [-17,0; 45,7] |  |
| **Joint symptoms** (*vs*. no symptoms) | -4,7 | [-32,5; 23,2] | 0,74 |
| **Muscle symptoms** (*vs*. no symptoms) | 37,0 | [-2,3; 76,4] | 0,07 |
| **Hemoglobin** (per 1-g/dL increment) | 7,2 | [-3,1; 17,5] | 0,17 |
| **CRP** (per 1-mg/L increment) | -0,1 | [-3,1; 2,9] | 0,96 |
| **Estimated GFR** (per 1-mL/min/1.73m^2^ increment) | 0,2 | [-0,4; 0,9] | 0,51 |
| **Initial HR** (per 1-bpm increment) | -1,3 | [-2,5; -0,1] | 0,03 |
| **ΔHR** (per 1-bpm increment) | 2,5 | [1,4; 3,5] | <10^-3^ |
| **LVEF** (per 1-% increment) | 1,6 | [0,0; 3,3] | 0,06 |
| **Positive chronotropic drug intake** (*vs*. no intake) | -4,4 | [-73,5; 64,8] | 0,90 |
| **Negative chronotropic drug intake** (*vs*. no intake) | -26,0 | [-55,9; 3,9] | 0,09 |
| **HAQ-DI score** (per 1-unit increment) | -77,1 | [-101,3; -53,0] | <10^-6^ |

N=142. R^2^=0.72, adjusted R^2^=0.67.

β-coefficients were expressed in meters.

6MWD: 6-minute walk distance; BMI: body mass index; CI: confidence interval; CRP: C-reactive protein; dc: diffuse cutaneous; GFR: glomerular filtration rate; HAQ-DI: Health Assessment Questionnaire-Disability Index; HR: heart rate; ILD: interstitial lung disease; lc: limited cutaneous; LVEF: left ventricle ejection fraction; PAH: pulmonary arterial hypertension; SSc: systemic sclerosis; Δ: variation of.
